# Effects of Laser Shock Processing on Morphologies and Mechanical Properties of ANSI 304 Stainless Steel Weldments Subjected to Cavitation Erosion

**DOI:** 10.3390/ma10030292

**Published:** 2017-03-15

**Authors:** Lei Zhang, Jin-Zhong Lu, Yong-Kang Zhang, Hai-Le Ma, Kai-Yu Luo, Feng-Ze Dai

**Affiliations:** 1 School of Food and Biological Engineering, Jiangsu University, Zhenjiang 212013, China; fdjy2017@gmail.com (L.Z.); mhl@ujs.edu.cn (H.-L.M.); 2 School of Mechanical Engineering, Jiangsu University, Zhenjiang 212013, China; kyluo@ujs.edu.cn (K.-Y.L.); dfz@mail.ujs.edu.cn (F.-Z.D.); 3 School of Electro-mechanical Engineering, Guangdong University of Technology, Guangzhou 510006, China; zykseu@163.com

**Keywords:** laser shock processing, cavitation erosion, laser weldment

## Abstract

Effects of laser shock processing (LSP) on the cavitation erosion resistance of laser weldments were investigated by optical microscope (OM), scanning electron microscope (SEM) observations, roughness tester, micro hardness tester, and X-ray diffraction (XRD) technology. The morphological microstructures were characterized. Cumulative mass loss, incubation period, erosion rate, and damaged surface areas were monitored during cavitation erosion. Surface roughness, micro-hardness, and residual stress were measured in different zones. Results showed that LSP could improve the damage of morphological microstructures and mechanical properties after cavitation erosion. The compressive residual stresses were generated during the process of LSP, which was an effective guarantee for the improvement of the above mentioned properties.

## 1. Introduction

The process of cavitation erosion is complicated and generated under the interaction of mechanical, chemical, and electrochemical functions [1]. The ultrasonic transducer sends out acoustic beams, and standing waves form via focusing. A temporary negative-pressure region is formed in a local area. Small cavitation bubbles in the unstable state are formed. At the same time, high pressure appears in the local area, and interface fracture is generated between liquids or solid liquid. Microwave is introduced through the growth, development, and collapse of the bubbles. The whole behavior of cavitation erosion is closely related to water hammer effect [2]. Under such circumstances, many mechanical components, such as steam turbine, blade, and storage tank, are destroyed by being subjected to cavitation erosion [3], especially for the surface of stainless steel weldments. As a result, mechanical properties of the weldments should be improved.

In fact, cavitation erosion is a type of dynamic damage, and the fatigue damage on the material surface is caused by the impact of cavitation bubbles. The distortion and erosion of the material is generated on the surface because of repetitive impact of cavitation bubbles. The material surface is then damaged by degradation, leading to the decrease in material service lives and the increment in maintenance cost [4]. Lei et al. investigated the cavitation erosion behavior of austenitic stainless steel. The surface of austenitic stainless steel was treated by weld cladding with the thickness of 3 mm, and the weld cladding layer was remelted by argon tungsten inert gas welding (TIG). The γ-austenite to ε-martensite phase transformation was induced by absorbing a significant amount of impact energies during cavitation erosion. Numerous small fragments formed by the fracture of martensitic plates were removed from the material surface. Finally, the incubation period of cavitation erosion ended [5]. The authors [6] studied the effects of cavitation erosion on a type of Fe-Cr-Ni-Co overlaying alloy with the thickness of 5 mm prepared by TIG on the surface of ANSI 304 stainless steel (ANSI 304 SS). The cavitation erosion resistance of the compound layer treated by the overlaying and remelting technology was better than that of the ANSI 304 SS base metal, and that of the remelting layer was better than that of the overlaying layer. Crack propagation was prevented by the refined grains during the TIG remelting process. Ju et al. [7] deposited a Pd-Ni/Pd-Cu double coating with the thickness of 3 μm on stainless steel surface by electroplating. Because of lower porosity, higher hardness, elasticity modulus and higher adhesive strength, the coating owned better corrosion resistance. Therefore, the main goals of the above researches are to strengthen the material surface and protect it from cavitation erosion. Chang et al. [8] recommended that the coating on the material surface should be thick enough to prevent corrosion erosion, and its thickness perhaps should be more than 10 μm.

In recent years, some other surface coating techniques have also been used to make material surface harden, such as surface mechanical attrition treatment (SMAT), ultrasonic impact peening (UIP), nitriding, and carburizing. Among these techniques, weld cladding, SMAT, and UIP have difficulty obtaining a coating at the nano-level on the surface of large size or complexly shaped parts [9]. Although nitriding and carburizing can solve this problem, they need the condition of high temperature, and the nanoparticles may fall off from the material surface. The coating may also come off. Laser shock processing (LSP) is known as an advanced surface modification technology. It is a flexible non-contact solid-state processing technique based on a shock wave. It can cause severe plastic deformation on the material surface in order to generate grain refinement and dislocation, and the depth of compressive residual stress is about 0.8 mm [9,10]. Thus, it can be adapted to various material systems at room temperature or other atmospheric environments. Moreover, it can prepare micro or nano coating on complex parts of any size. The strength and characteristics of materials, such as residual stresses and hardness [11], wear and friction properties [12], and corrosion resistance [13,14], can be improved to a certain extent through LSP. The process of LSP acting on the material surface is described in detail [15,16,17,18], and the effects of LSP on stainless steel were discussed by our research group.

In the current work, LSP treatment is applied to improve the cavitation erosion resistance of laser weldments. The surface of ANSI 304 SS thick sheets treated by laser welding was irradiated during LSP. The experiment results were explained through the analysis of morphological microstructures and mechanical properties. The surface morphologies of the samples were visually displayed by optical microscope (OM) and scanning electron microscope (SEM), because it is necessary to develop a comprehensive knowledge on the microstructure modification mechanisms systematically [19]. At the same time, the mechanical properties of the samples were measured, such as hardness. Hattori et al. [20] has reported the relation between the erosion resistance and hardness. The higher the hardness is, the stronger the erosion resistance is. In this way, the effects of LSP on the resistance to cavitation erosion were studied from outward appearance to inner essence.

## 2. Materials and Methods

### 2.1. Materials and Laser Welding Procedure

ANSI 304 SS sheets with a thickness of 5 mm were used as the welding material. The chemical composition of this material is given in Table 1.

**Figure 1:**
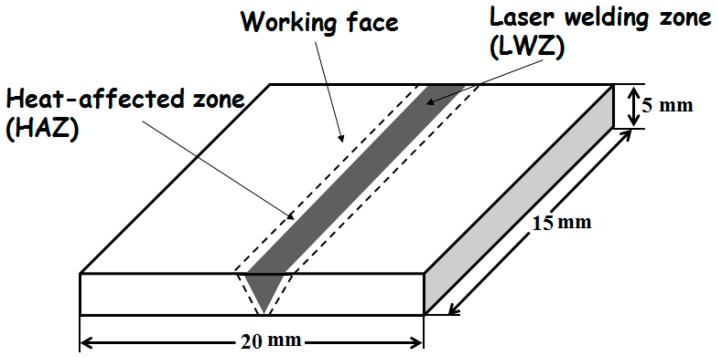
Schematic diagram of laser weldment.

The above-mentioned materials were treated using YLS−4000 fibre laser (IPG, Oxford, MA, USA). The butt weld was processed with full penetration. The laser welding parameters were selected and shown in Table 2. The spot diameter of the laser beam was 0.27 mm with a focal length of 190 mm. Ultra-high mixed gas integrating helium (He) and argon (Ar) (1:1 mixing ratio) was used as the shielding gas during laser welding. The flow rate of adding gas was 0.6 m^3^/h in the condition of coaxial adding gas. The LWZ was located in the middle of the laser weldment. The laser welding surface was smooth without spatter and distortion because of high energy density and low heat input procedure. The width of the HAZ was small, as seen in Figure 1. The area of the working face was 20 mm × 15 mm.

### 2.2. Laser Shock Processing Experiment

The working face of the laser weldment was polished with SiC paper with different grades of roughness (from 150# to 1600#) before LSP, followed by cleaning in deionised water. Subsequently, the laser weldment was degreased in ethanol by ultrasonic cleaning. Then, the surface of the laser weldment was treated by a GAIA-R Q-switched Nd: YAG laser (THALES, Paris, France) with a wavelength of 1064 nm and a pulse duration of about 15 ns. After a lot of experiments for optimizing process parameters, the laser parameters were determined [21,22]. The repetition rate was 1 Hz, laser spot diameter was 3 mm, and overlapping rate was 50% between two adjacent spots to ensure no blind area at the LSPed region. The laser pulse energy was 6 J. Because of stickiness itself, a 0.1 mm-thick aluminium foil was compactly joined on the surface of the laser weldment as an absorbing layer to improve the absorption from laser pulse energies and protect the material surface from laser ablation. A water layer with a thickness of 1–2 mm was used as the transparent confining layer to increase the peak pressure of laser shock waves, the impulse in the samples and the action time [23]. Finally, the aluminium foil was torn off from the surface and the samples were cleaned in deionised water and degreased in ethanol by ultrasonic cleaning again.

### 2.3. Cavitation Erosion Experiment

**Figure 2:**
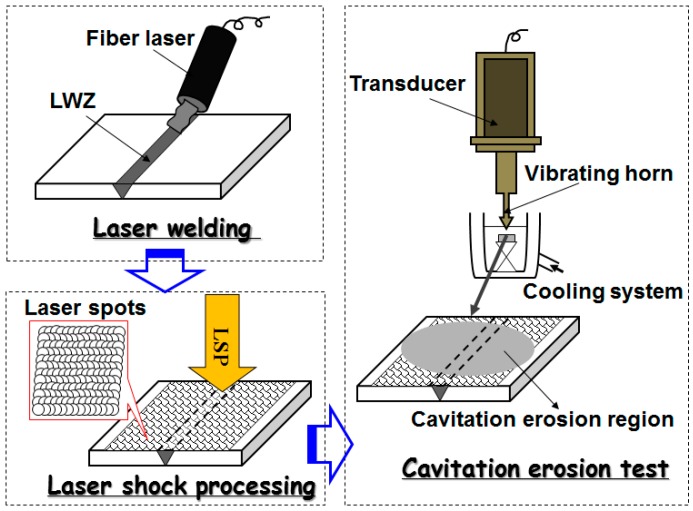
The process sequence graph (PSG).

The LSPed samples were polished before cavitation erosion test to avoid the interference of micro-indents by LSP. Cavitation erosion experiment was carried out in distilled water using an ultrasonic vibratory facility with a vibrating horn, according to ASTM G32−09 standard [24]. The testing parameters are presented in Table 3. The samples were subjected to cavitation erosion tests. The tip of the vibrating horn was submerged in water and aimed precisely at the middle of the samples containing the LWZ and HAZ. According to instructions of this facility, the distance between the samples and the vibrating horn tip was 10 mm best. The cavitation erosion test lasted for at least 6 h. The process sequence graph of the above-mentioned experimental procedures, including laser welding, LSP, and cavitation erosion, is given in Figure 2.

### 2.4. Morphological Observation

Morphologies on the surface of the samples were characterised by using a DM 2500 M optical microscope (OM) (LEICA, Wetzlar, Germany). Thereinto, the original surface morphology of weldment should be corroded in the aqua regia for several seconds before OM observation, while the surface morphologies of the samples could be observed directly after cavitation erosion. All the surface of the samples should be covered with gold before the observation of JSM−7001F scanning electron microscope (SEM) (JEOL, Tokyo, Japan).

### 2.5. Test Exposure to Ultrasound Cavitation Ageing

After cavitation erosion experiment, the samples were degreased, rinsed, dried, and weighed using an analytical high-precision electronic balance with an accuracy of 0.1 mg at room temperature. The mass of samples was monitored at regular intervals of 0.5 h, and the total erosion time was 6 h. Finally, the cumulative mass loss was derived. The final damaged surface areas of the samples were measured using a vernier calliper after cavitation erosion.

### 2.6. Surface Roughness, Micro-Hardness, and Residual Stress Measurement

The surface roughness in the LWZ and HAZ of the samples were measured using a 130A roughness tester (Surfcom, Tokyo, Japan) during cavitation erosion.

The cross-sectional hardness of the samples was analysed by a HXD−1000TMSC/LCD micro hardness tester (Shanghai CaiChen Precise Instrument Co., Ltd., Shanghai, China) using a diamond indenter (NO. HV 110716) at room temperature. The hardness gauge was HV, the multiplying power of the objective lens was 40×, the force was 1.961 N (200 gf), the hold time of the force was 15 s, and the surface shape was plane.

The X-ray diffraction (XRD, ST, Han Dan, China) method known as the sin2ψ method was applied to measure residual stresses on the surface of the LWZ and HAZ of the samples. The X-ray source was the Cr-Kβ X-ray with a beam diameter of 1 mm, and the diffraction plane was β phase (311) plane. The speed of ladder scanning was 0.1%/s. The scanning starting angle and terminating angle were approximately 145° and 153° respectively. Poisson’s ratio was set to 0.35. Measurements were repeated three times for each condition and the average values were obtained.

## 3. Results and Discussion

### 3.1. Surface Morphologies Observed under Different Ways and Magnifications

#### 3.1.1. Morphological Observation by OM

**Figure 3:**
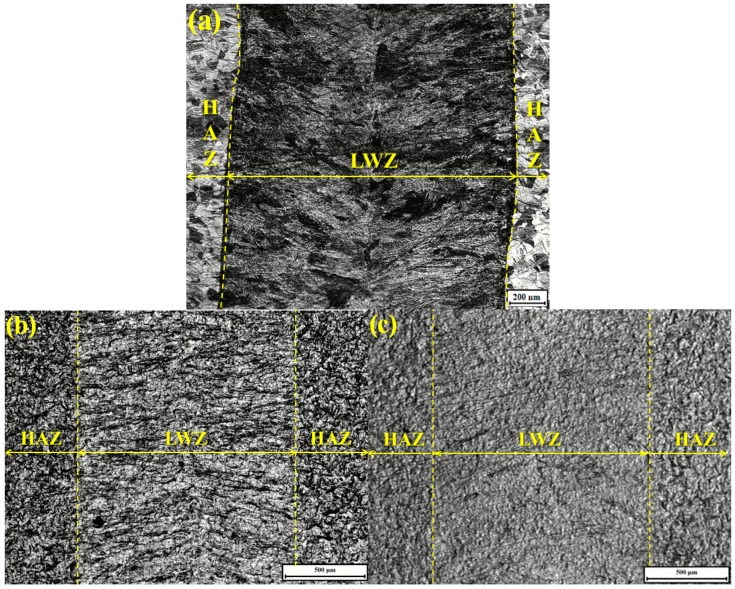
Typical optical microscope (OM) micrographs of surface morphologies: (**a**) Original surface morphology of weldment; (**b**) Untreated sample after cavitation erosion; (**c**) LSPed sample with 6 J pulse energy after cavitation erosion.

Figure 3a gives the original surface morphology of weldment without any treatment. It looks like fish-bone; the crystal solidified microstructure is dendrite and grows with opposite orientations from the middle of the laser welding zone (LWZ). The crystal microstructure of the heat−affected zone (HAZ) is polygonal. All morphologies in the LWZ and HAZ are in the same level surface. Figure 3b,c shows the respective surface morphologies of the untreated and LSP shocked (LSPed) samples after cavitation erosion. The surface morphologies are in an uneven state due to the eroded process of cavitation erosion layer by layer. Surface morphologies in the LWZ are different from those in the HAZ. Undulations and upheavals that present a dendrite structure are observed on the surface of the LWZ, whereas common undulations and upheavals appear on the surface of the HAZ. The degree of undulations and upheavals is higher in the HAZ than that of the LWZ because the surface of the HAZ is most likely to be damaged by cavitation erosion [25]. The degree of damage on surface morphologies in Figure 3b is deeper and denser than that in Figure 3c. In short, the surface of untreated samples is more prone to damage by cavitation erosion compared with that of the LSPed samples.

**Figure 4:**
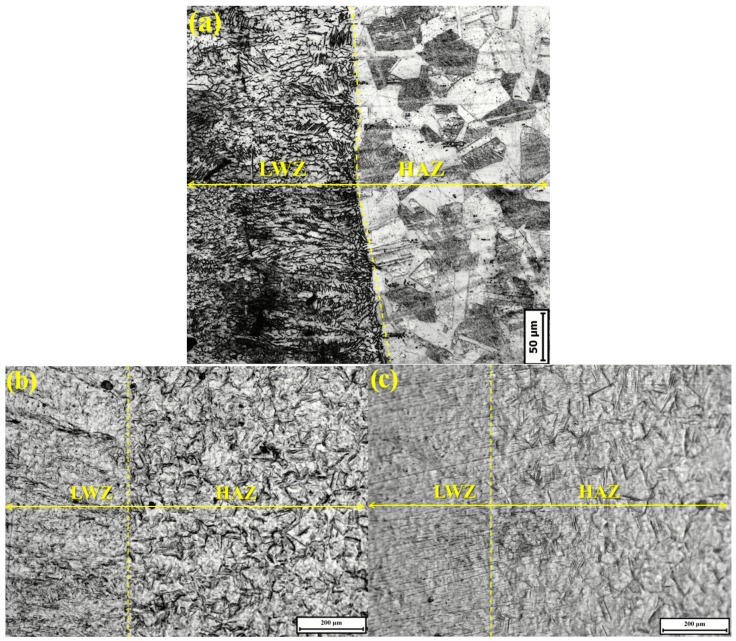
High magnification OM micrographs of surface morphologies in the heat-affected zone (HAZ) from Figure 3: (**a**) Original surface morphology of weldment; (**b**) Untreated sample after cavitation erosion; (**c**) LSPed sample with 6 J pulse energy after cavitation erosion.

Figure 4a gives high magnification OM micrograph of the original surface morphology of weldment without any treatment. From it, polygonal crystal microstructure can be seen clearly in the HAZ. Figure 4b,c is high magnification OM micrographs of surface morphologies in the HAZ from Figure 3b,c. Undulations and upheavals are found to begin at the twin boundaries. The number of delineated twin boundaries and small pits in Figure 4b is higher than that in Figure 4c. This outcome indicates that the surface of the HAZ of the untreated samples is more prone to damage by cavitation erosion than that of the LSPed ones. Laser pulse energies can improve the cavitation erosion resistance in the HAZ of the samples more efficiently, which increases the overall properties of laser weldments.

Combined with Figure 3 and Figure 4, three conclusions can be drawn: first, the surface morphologies in the LWZ are different from those in the HAZ; second, the original morphologies are in the same level surface, but those become uneven after cavitation erosion and undulations and upheavals appear with different shapes; and third, the degree of undulations and upheavals of the LSPed samples becomes shallower than that of the untreated ones.

#### 3.1.2. Morphological Observation by SEM

**Figure 5:**
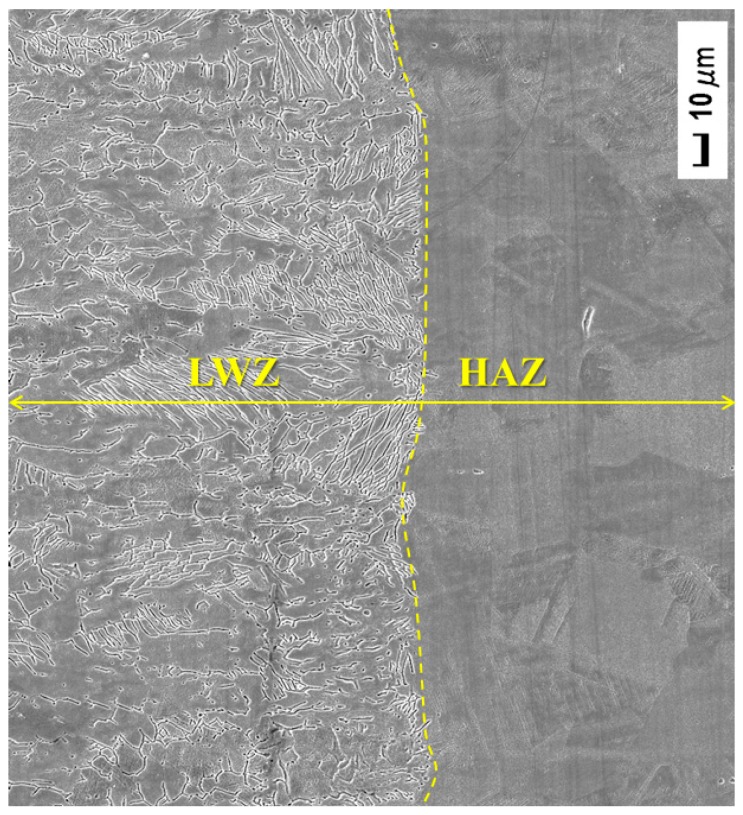
Scanning electron microscope (SEM) observation of surface morphology of untreated laser weldment before cavitation erosion.

Figure 5 shows gold-sprayed SEM micrograph of the surface morphology of untreated laser weldment before cavitation erosion. The surface morphology is flat and undamaged without pits. The crystal microstructure of LWZ is dendrite and that of the HAZ is polygonal, which is the same as Figure 3a and Figure 4a.

**Figure 6:**
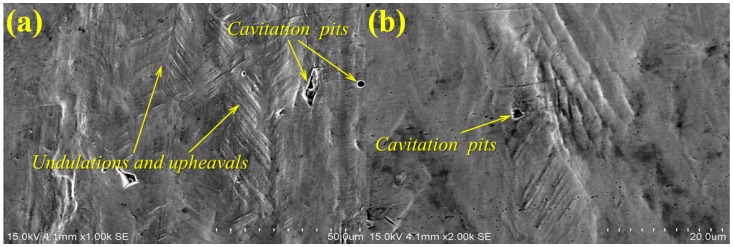

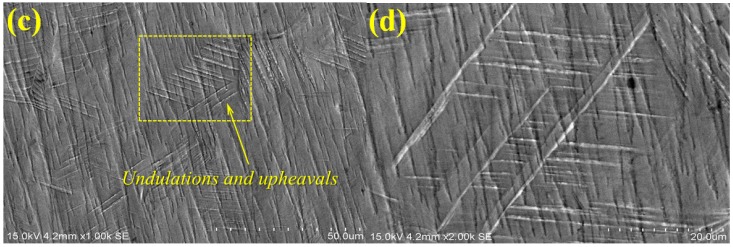
Typical SEM observation of surface morphologies in the laser welding zone (LWZ) after cavitation erosion: (**a**) Untreated sample; (**b**) High magnification SEM micrograph of (a); (**c**) LSPed sample with 6 J pulse energy; (**d**) High magnification SEM micrograph of (c).

Figure 6 shows the SEM micrographs of the surface morphologies in the LWZ of the samples after cavitation erosion. Undulations and upheavals that present a dendrite structure can be observed. Figure 6a,b indicates that undulations and upheavals are more obvious and denser on the surface of the LWZ of the untreated samples. Particles are removed from the surface of the LWZ, and some cavitation pits appear because of the impact of cavitation bubbles. However, undulations and upheavals in the LWZ of the LSPed samples are shallower and sparser, as shown in Figure 6c,d. Given that repetitive impacts are generated by the collapse of cavitation bubbles, the impact energies can cause plastic deformation (microscopic deformation) and fracture. Highly concentrated stresses also appear easily in the samples with low hardness, leading to cracks and removal of materials from the surface after cavitation erosion [26,27]. When absorbing more impact energies of cavitation bubbles is difficult for the macroscopic deformation, the generation of cracks and fracture and the removal of materials are restrained. The best way to absorb the impact energies of cavitation bubbles is through slip or phase transformation, which can appear because of LSP [28]. The dislocations move along the slip surfaces, causing the generation of dislocation intersections and the appearance of dislocation tangles, thus inducing surface hardening. The dislocation motion encounters stronger resistance stress, and the stronger applied stresses will be used to induce deformation [29]. Consequently, undulations and upheavals on the surface of the LSPed samples are difficult to occur.

**Figure 7:**
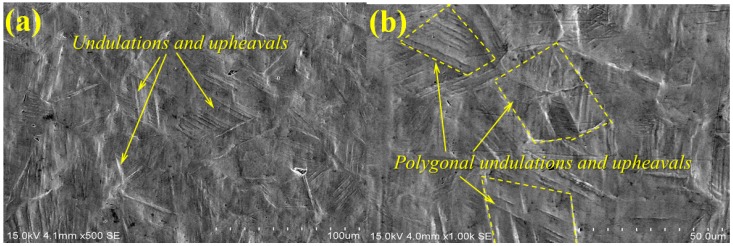

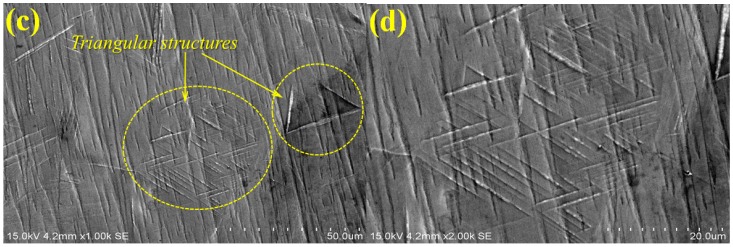
Typical SEM observation of surface morphologies in the HAZ after cavitation erosion: (**a**) Untreated sample; (**b**) High magnification SEM micrograph of (a); (**c**) LSPed sample with 6 J pulse energy; (**d**) High magnification SEM micrograph of (c).

Figure 7 shows the SEM micrographs of surface morphologies in the HAZ of the samples after cavitation erosion. They are different from those in the LWZ in Figure 6. Owing to the repetitive impact of cavitation bubbles, polygonal undulations and upheavals appear on the surface of the HAZ. From Figure 7a,b, the undulations and upheavals in the HAZ of the untreated samples are more obvious and deeper compared with those of the LSPed ones, as shown in Figure 7c,d. The edges of the polygonal undulations and upheavals are distorted severely and connected to form numerous of cleavage steps, as shown in Figure 7a,b, but almost not in Figure 7c,d. The effects of compressive stresses result in the formation of triangular structures along the slip, as shown in Figure 7c,d. The formation of triangular structures is related to the phase transformation from γ-austenite to ε-martensite along the austenite plane {111} [5]. According to the hard-sphere model, phase transformation is only half of the external stacking fracture energy. This transformation may absorb considerable amounts of impact energies from cavitation bubbles, resulting in the decrease of crack generation and propagation [30,31]. The phase transformation of the martensite in the material can be caused by the LSP itself [32]. Moreover, high-level compressive residual stresses are generated in the laser strengthening layer of the LSPed samples, which cause the material surface to harden and mechanical properties to strengthen. Thus, the distortion and fracture of martensitic plates are restrained effectively, and the generation and propagation of cracks are restrained further. The LSPed samples obtain better resistance to cavitation erosion.

### 3.2. Mechanical Properties Measured during Cavitation Erosion

#### 3.2.1. Measurement in the Development Process of Cavitation Erosion

**Figure 8:**
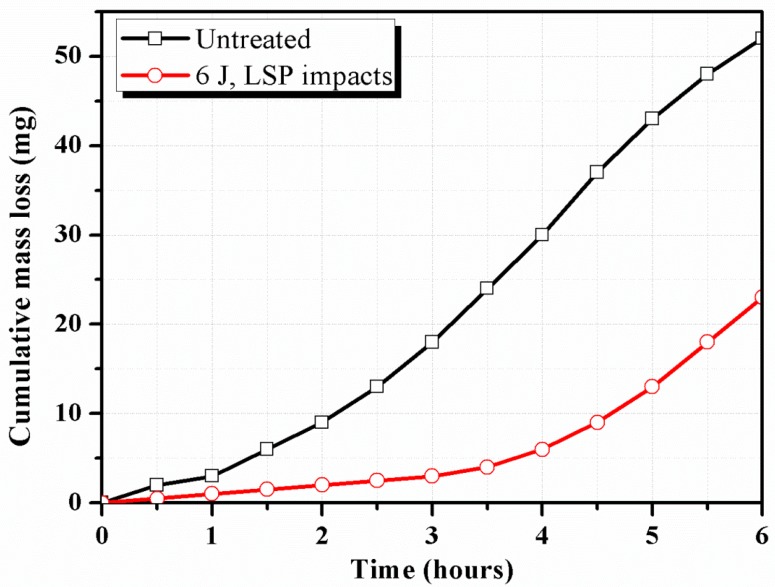
Cumulative mass loss of the samples after cavitation erosion.

Figure 8 shows the cumulative mass loss of the samples as a function of cavitation erosion time. The mass of the samples is monitored according to ASTM G32−09 standard at regular intervals of 0.5 h [24]. In general, the cumulative mass loss increases with the increment of erosion time. The curve of the LSPed samples is lower and varies more slowly than the untreated ones. The maximum mass loss is 50.0 mg for the untreated ones after an erosion time of 6 h, whereas it is only about 23.0 mg for the LSPed ones.

**Figure 9:**
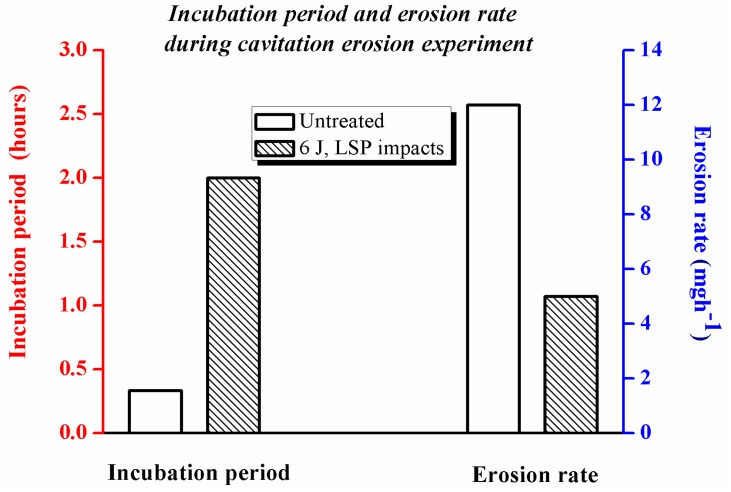
Incubation period and maximum erosion rate reported during the experiment.

With respect to the incubation stage, a longer incubation period (2 h) is observed for the LSPed sample than for the untreated one (1/3 h), as shown in Figure 9. The incubation period is calculated following the ASTM G32 standard as the intercept on the testing time axis of a straight-line extension of the maximum-slope portion of the cavitation erosion time curve [24]. Figure 9 also illustrates the maximum erosion rate reported during the tests for all samples, calculated from the time-variation cumulative mass curves presented in Figure 8. The untreated samples show a higher erosion rate than the LSPed ones, reaching as high as 2.5 times, which aggravates the cavitation erosion of laser weldments. Thus, the LSPed samples exhibit more excellent resistance to cavitation erosion than the untreated ones.

**Figure 10:**
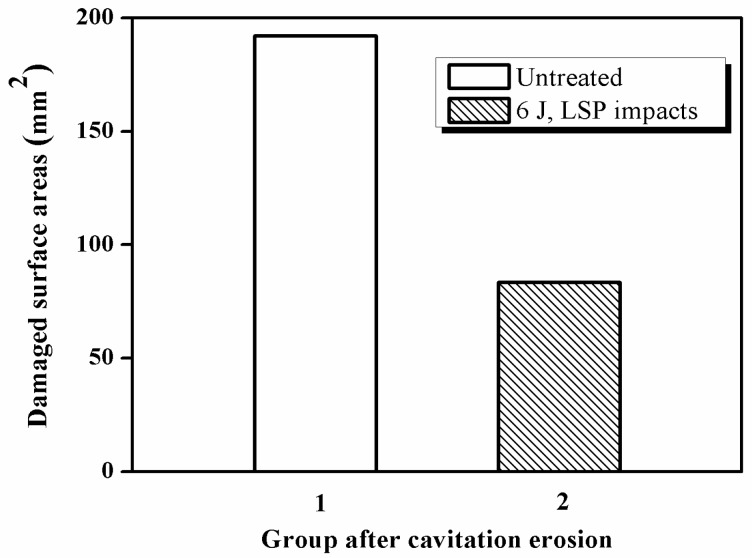
Damaged surface areas of the samples after cavitation erosion.

Figure 10 shows the damaged surface areas of the samples after cavitation erosion. The damaged surface areas of the untreated and LSPed samples are 192.1 and 83.5 mm^2^ respectively. At the same time, the ratios of their damaged surface area to the working face are 64.0% and 27.8% respectively. Therefore, we can deduce that the cavitation erosion resistance is improved by LSP.

#### 3.2.2. Surface Roughness, Micro-Hardness, and Residual Stress Analysis

**Figure 11:**
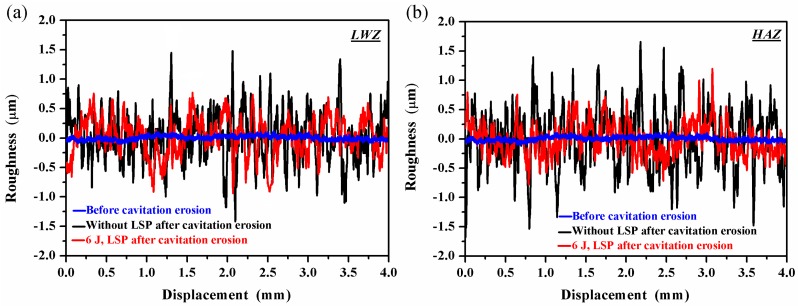
Roughness profiles of the untreated and LSPed samples before and after cavitation erosion: (**a**) In the LWZ; (**b**) In the HAZ.

Figure 11 shows the typical surface roughness profiles of the samples before and after cavitation erosion. Figure 11a presents the surface roughness profiles in the LWZ. The surface roughness of the LSPed samples is shown to be lower than that of the untreated ones. Figure 11b presents the surface roughness profiles in the HAZ. The surface roughness in the HAZ of the untreated samples is higher than that of the LSPed ones. A comparison of Figure 11a,b reveals that the surface roughness in the LWZ is a little lower than that in the HAZ, and all the roughness in the LWZ and HAZ is higher after cavitation erosion, as listed in Table 4. It is indicated that the surface of samples is damaged by cavitation effect. However, local stress concentrations will be generated because of the higher surface roughness, which leads to the initiation and growth of fatigue cracks [33]. Crack initiation is an important period during fatigue crack propagation, which directly determines the fatigue failure. The surface of the samples is in the polished state at first and is then destroyed after cavitation erosion. The surface roughness increases with the increment of time. The higher the surface roughness, the worse the cavitation erosion resistance [34,35]. Given that the HAZ becomes a vulnerable area, cavitation erosion occurs easily here [33]. However, the surface roughness of the samples, including the HAZ, decreases because of the effects of LSP, which improves all the mechanical properties of the samples. Thus, the cavitation erosion resistance of the samples is improved through LSP.

**Figure 12:**
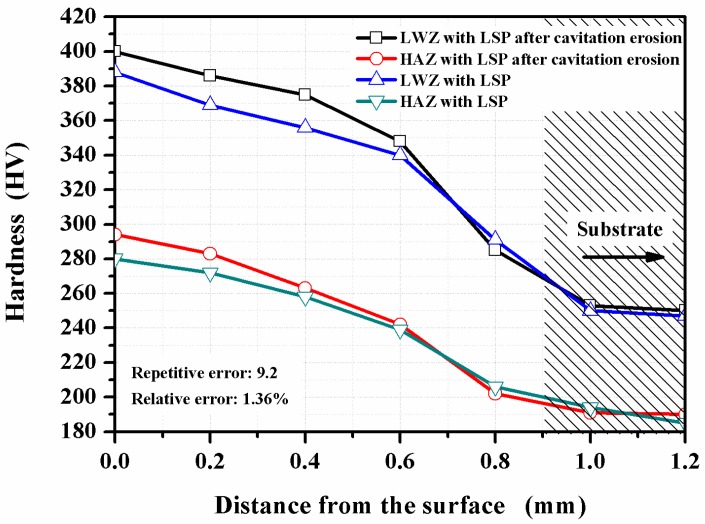
Cross-sectional hardness of the LSPed samples.

Figure 12 gives the cross-sectional hardness in the LWZ and HAZ of the LSPed samples before and after cavitation erosion. The effects of LSP on the hardness distribution of cross sections are remarkable, and the hardness value is proportional to the LSPed effects. The hardness value is the highest on the LSPed surface, but decreases gradually with the increment in depth. When beyond the depth of 0.8−1.0 mm, it reaches a stable value that belongs to the substrate (untreated zones). The change in trend of the cross-sectional hardness has a close relation to the distribution of compressive residual stress and is even similar to it [14]. Compared with the substrate, the hardness values increase respectively by 57% and 51% on the LSPed surface in the LWZ and HAZ before cavitation erosion. In addition, they increase respectively by 60% and 55% on the LSPed surface in the LWZ and HAZ after cavitation erosion. On the other hand, the average values of the cross-sectional hardness in the LWZ are higher than that in the HAZ and the substrate. The solidified crystal microstructure of the LWZ is dendrite, which is completely different from those of the HAZ and the substrate. The alloy elements in the LWZ have no time to form the second phase to precipitate because of the high cooling speed during the laser welding procedure. These elements dissolve to a great degree. When a certain amount of ferrite with a dendritic structure occurs in the LWZ, grains become fined. Thus, the LWZ exhibits the highest micro-hardness values [36]. The increment in hardness is also the main precondition for improving the cavitation erosion resistance [37].

Table 5 shows the average values of surface residual stress in the LWZ and HAZ of the samples. For the untreated samples, the average values of surface residual stress in the LWZ and HAZ are −5 and 21 MPa respectively, which are almost in a state of tensile residual stress, and the LWZ trends toward the compressive residual stress. In comparison, the LSPed surface in the LWZ and HAZ is in a state of high-level compressive residual stress, and the absolute value in the LWZ is higher than that in the HAZ. In conclusion, high-level compressive residual stress is induced by laser shock waves on the surface of the samples during LSP. The compressive residual stress mainly gathers on the surface or near-surface zones of the materials. This layer, which is named as the laser strengthening layer, has the affected depth of approximately 0.8−1.0 mm. Tensile residual stress deteriorates material mechanical properties and accelerates its fatigue failure from erosion and corrosion. On the contrary, compressive residual stress on the surface of materials can delay crack initiation and slow the growth of micro-cracks [38,39]. Hence, cavitation erosion resistance can be improved because of the existence of compressive residual stress during LSP.

### 3.3. Strengthening Mechanism Explain of Cavitation Erosion Resistance during LSP

**Figure 13:**
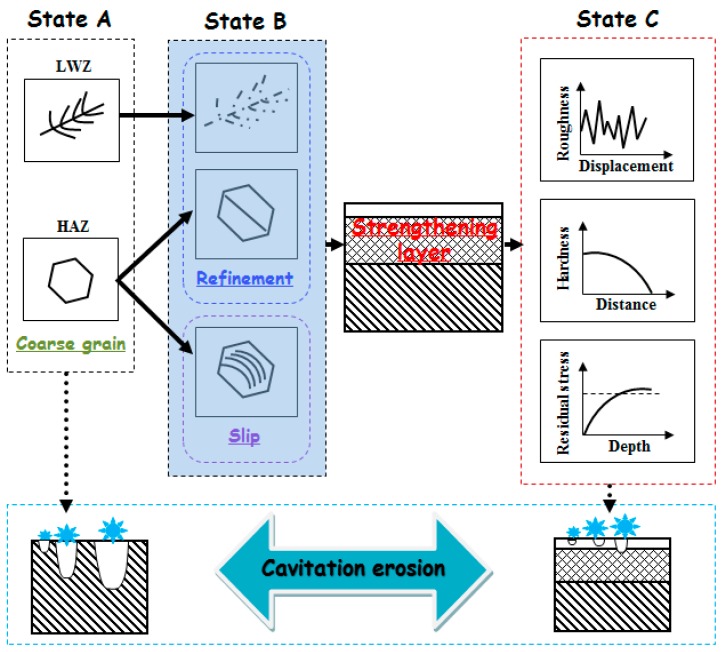
Schematic illustration showing the improvement of the cavitation erosion resistance by LSP.

Figure 13 shows the improvement process of the cavitation erosion resistance by LSP. The original microstructure is ferrite with a dendrite structure in the LWZ, that is mainly austenite with a polygonal figure in the HAZ, and the laser weldment is made of ANSI 304 stainless steel in “State A”, as seen in Figure 13. The original microstructure in the LWZ can be easily distinguished from that in the HAZ in Figure 3a, Figure 4a and Figure 5. After cavitation erosion, the undulations and upheavals that present a “tree branch-like” dendrite structure are observed in the LWZ in Figure 3b. By comparison, common polygonal undulations and upheavals appear on the surface in the HAZ in Figure 4b. However, Figure 13 illustrates that their surface morphologies are seriously destroyed because of cavitation erosion, which is their common point.

LSP is a green and advanced surface modification technology with a high peak pressure of laser shock waves (up to the GPa level). To a certain extent, the effect of LSP is similar to a sledgehammer knocking on the surface of the materials. As a result, the material surface becomes compact and smooth. The strengthening layer (mm level) on the material surface can be formed. The grain refinement (in ‘State B’ shown in Figure 13) occurs in this strengthening layer, which is different in the LWZ and the HAZ. The process of grain refinement is very complex. ANSI 304 stainless steel belongs to the face-centred cubic (fcc) materials. The grain refinement is induced by plastic strain with low stacking fault energy during LSP. Prof. Lu [23] proposed the process of grain refinement along the depth direction by LSP. First, planar dislocation arrays and stacking faults are formed because of the pile up of dislocation lines during LSP. Second, sub-micron triangular blocks appear because of multi-directional mechanical twin matrix (MT)–MT intersections. Third, MT is transformed into sub-grain boundaries. Finally, the refined grain boundaries are formed because of continuous dynamic recrystallisation of sub-grain boundaries driven by LSP. “State B” in Figure 13 also shows that the slip system is found in the austenite grains after LSP. The material surface is affected by LSP, and the surface moves outwardly. At the same time, compressive residual stresses equal to the shear stresses are generated in the near-surface regions. The micro-steps occur under the effects of their extrusion. Slip systems are then formed. The slips are used to absorb the impact energies of cavitation bubbles and cooperate with the dislocation movement through LSP [28,40]. Cavitation erosion can hardly occur with relatively fewer impact energies of cavitation bubbles. Thus, the cavitation erosion resistance of the material is improved because of LSP.

Based on the above mentioned process caused by the effects of LSP, the mechanical properties of the material are improved in “State C” as shown in Figure 13. The surface roughness of the material decreases (Figure 11), crack initiation is restrained effectively on the surface and resistance to cavitation erosion is strengthened. The micro-hardness of the material is improved within the depth of 0.8−1.0 mm (as shown in Figure 12). Thus, the material surface is difficult to corrode layer by layer during the process of cavitation erosion. The high-level compressive residual stresses are induced with the help of LSP (as shown in Table 5). When the material surface is irradiated by the laser, the subsurface material is subjected to elastoplastic waves, which generate uniaxial plastic strain. The surrounding material then moves along the opposite direction of the strain, and biaxial compressive residual stresses are generated in the strengthening layer [41], which can restrain the initiation and growth of cracks during cavitation erosion.

## 4. Conclusions

Many engineering materials are destroyed because of cavitation erosion, including laser weldments made of ANSI 304 stainless steel. In this research, the cavitation erosion behaviour of materials was observed. Their morphological microstructures and mechanical properties were compared. Several important conclusions can be drawn, as follows:

(1) The surface morphologies of the samples are damaged after corrosion erosion. Those in the LWZ are different from that in the HAZ as demonstrated through the analysis of OM and SEM observations. Undulations and upheavals that present a dendrite structure occur on the surface of the LWZ, whereas common undulations and upheavals appear on the surface of the HAZ. All these undulations and upheavals become shallower and sparser because of LSP. The degree of damage on the surface morphology is relieved by LSP.
(2) Compared with the untreated samples after cavitation erosion, the cumulative mass loss, erosion rate, and damaged surface areas decrease through LSP, whereas the incubation period become longer for LSPed samples. The surface roughness decreases through LSP after cavitation erosion. The surface roughness in the LWZ is lower than that in the HAZ. The hardness value is at its highest on the surface through LSP. The cross-sectional hardness in the LWZ is higher than that in the HAZ for the samples. Moreover, the LSPed surface of the LWZ and HAZ is in a state of high-level compressive residual stress, and the absolute value in the LWZ is higher than that in the HAZ. Hence, cavitation erosion resistance can be improved because of the existence of compressive residual stress.

## Figures and Tables

**Table 1:** Chemical composition of ANSI 304 SS (wt %).

C	Si	Mn	Cr	Ni	S	P
≤0.08	≤1.0	≤2.0	18.0−20.0	8.0−10.0	≤0.03	≤0.035

**Table 2:** Welding parameters selected in the present study.

Laser Type	Laser Power P (kW)	Welding Speed V (mm·s^–1^)	Defocusing Distance Δf (mm)
Fiber laser	4	30	−3

**Table 3:** Testing parameters in cavitation erosion tests.

Diameter of Vibrating Horn (mm)	Frequency (kHz)	Peak-to-Peak Amplitude (µm)	Temperature (°C)	Total Testing Time (h)
12	19−21	50	20	At least 6

**Table 4:** Value of roughness before and after cavitation erosion.

Sample	Ra (μm)	
	LWZ	HAZ
Before cavitation erosion	0.02	0.02
Without LSP after cavitation erosion	1.26	1.37
6 J, LSP after cavitation erosion	0.68	0.89

**Table 5:** The average values of surface residual stress in the LWZ and HAZ of the samples.

Caption	The Value of Surface Residual Stress (MPa)	
	LWZ	HAZ
Untreated	−5	21
6 J, LSP impacts	−402	−335
